# Carotenoids and Their Isomers: Color Pigments in Fruits and Vegetables

**DOI:** 10.3390/molecules16021710

**Published:** 2011-02-18

**Authors:** Hock-Eng Khoo, K. Nagendra Prasad, Kin-Weng Kong, Yueming Jiang, Amin Ismail

**Affiliations:** 1 Department of Nutrition and Dietetics, Faculty of Medicine and Health Sciences, Universiti Putra Malaysia, 43400 UPM Serdang, Selangor, Malaysia; E-Mails: hockeng_khoo@yahoo.com (H.-E.K); knag76@gmail.com (K.N.P); kchai21@yahoo.com (K.-W.K); 2 South China Botanical Garden, Chinese Academy of Sciences, Guangzhou 510650, China; E-Mail: ymjiang@scbg.ac.cn (Y.J.); 3 Laboratory of Analysis and Authentication, Halal Products Research Institute, Universiti Putra Malaysia, 43400 UPM Serdang, Selangor, Malaysia

**Keywords:** carotene, cryptoxanthin, fruit, lutein, lycopene, vegetables, zeaxanthin

## Abstract

Fruits and vegetables are colorful pigment-containing food sources. Owing to their nutritional benefits and phytochemicals, they are considered as ‘functional food ingredients’. Carotenoids are some of the most vital colored phytochemicals, occurring as all-*trans* and *cis*-isomers, and accounting for the brilliant colors of a variety of fruits and vegetables. Carotenoids extensively studied in this regard include β-carotene, lycopene, lutein and zeaxanthin. Coloration of fruits and vegetables depends on their growth maturity, concentration of carotenoid isomers, and food processing methods. This article focuses more on several carotenoids and their isomers present in different fruits and vegetables along with their concentrations. Carotenoids and their geometric isomers also play an important role in protecting cells from oxidation and cellular damages.

## 1. Introduction

Increasing interest in nutrition, fitness and beauty consciousness has enhanced concerns over a healthy diet. Fruits and vegetables have assumed the status of ‘functional’ foods, capable of providing additional health benefits, like prevention or delaying onset of chronic diseases, as well as meeting basic nutritional requirements. Appropriate intake of a variety of fruits and vegetables ensures sufficient supply of nutrients and phytochemicals such as carotenoids. Low consumption of fruit and vegetable is among the top ten risk factors resulting in the global mortality. Annually, 2.7 million lives could be saved with sufficient consumption of various kinds of fruits and vegetables [1]. 

Nowadays, food scientists have collaborated with nutrition researchers to develop plant-based functional foods to promote healthy eating habits. In food research, carotenoids from fruits and vegetables have attracted a great deal of attention, mainly focused on the analysis of geometric carotenoid isomers. Carotenoids found in fruits and vegetables have also attracted great attention for their functional properties, health benefits and prevention of several major chronic diseases [2,3,4]. 

Carotenoids are synthesized in plants but not in animals. In nature, more than 600 types of carotenoid have been determined. Carotenoids are localized in subcellular organelles (plastids), *i.e.* chloroplasts and chromoplasts. In chloroplasts, the carotenoids are chiefly associated with proteins and serve as accessory pigments in photosynthesis, whereas in chromoplasts they are deposited in crystalline form or as oily droplets [5]. Some of the carotenoids such as the xanthophylls are involved in photosynthesis by participating in energy transfer in the presence of chlorophyll in plants [6]. 

Studies have shown that carotenoids contribute to the yellow color found in many fruits and vegetables [5,7]. The colors of fruits and vegetables depend on conjugated double bonds and the various functional groups contained in the carotenoid molecule [8]. A study also reported that the greater the number of conjugated double bonds, the higher the absorption maxima (λ_max_) [9]. As a result, the color ranges from yellow, red to orange in many fruits and vegetables [5,10]. Besides, esterification of carotenoids with fatty acids can also occur during fruit ripening, which may affect the color intensity [11]. 

Naturally, most of the carotenoids occur as *trans*-isomer in plants. However, *cis*-isomers may increase due to the isomerization of the *trans*-isomer of carotenoids during food processing [12]. Many studies have involved in the analysis of dietary carotenoids and their potential isomers [13,14,15], with much attention given to the geometric isomerization of carotenoids [16,17,18,19,20,21]. The investigation of carotenoid contents in fresh, frozen and canned foods has been carried out [22]. However, a recent review on contents of carotenoids and their isomers from diverse fruits and vegetables has not been made. The data collected from published literatures will be useful for food researchers, nutritionists and health practitioners in promoting right diets to minimize vitamin A deficiency and maintaining a healthy dietary practice.

## 2. Carotenoids and Their Isomers

There are many factors influencing the formation and isomerization of carotenoids. Heat, light, and structural differences are the prominent factors that affect the isomerization of carotenoids in foods [23,24,25]. Various processing methods, such as heating and drying also lead to the isomerization and even degradation of carotenoids [26,27]. De Rigal *et al*. [24] reported that isomerization of carotenoids in apricot purees was due to enzymatic browning. Oxidative degradation of carotenoids has also led to *cis*-*trans* isomerization and formation of carotenoid epoxides [28,29]. 

Previous studies have shown that *cis*-isomer of carotenoids can be identified based on the absorption spectrum characteristics, *Q* ratios, and the relative intensity of the *cis* peak [8,30]. The UV spectrum of *cis* carotenoids is characterized with their λ_max_ between 330–350 nm, which has greatest intensity when the double bond is located near or at the center of the chromophore [31]. On the other hand, a hypsochromic shift in the λ_max_ and smaller extinction coefficient is observed. Thus, *cis*-*trans* isomerization of carotenoids leads to a decrease of color intensity [12]. 

Carotenoids that contain more than seven conjugated double bonds were reported to have stronger antioxidant capacity and protection against photo-bleaching of chlorophyll [32]. Di Mascio *et al*. [33] also reported ^1^O_2_ quenching capability of carotenoids is based on the number of conjugated double bonds and not the ionone ring of β-carotene. As the geometric isomers of carotenoids make great contribution to antioxidant activities and health improvement, analyses of carotenoids and their isomers in fruits and vegetables are needed.

Liquid chromatography (LC) enables separation and identification of individual carotenoids. Identification of carotenoid isomers can be achieved by high performance liquid chromatography (HPLC). The separation of carotenoid isomers can be done using either polymeric C_30_ or ODS-2 silica columns [34]. However, the identification of carotenoid isomers seemed to be ambiguous. In this review, the analyses of carotenoid geometric isomers and their levels are listed in Table 1, which also should enable researchers to understand the various carotenoid isomers present in different fruits and vegetables. 

**Table 1:** Analyses of carotenoid isomers in fruits and vegetables.

Fruit/ vegetable	Analytical method	Carotenoid and its isomer		Ref.
Bambangan (lyophilized pulp) [ *Mangifera pajang* Kosterm.]	HPLC: Polymeric C30 column (150 mm × 4.6 mm i.d., 3 μm particle)	cryptoxanthin (mg/100 g): 1.18α-carotene (mg/100 g): all- *trans* (7.96)β-carotene (mg/100 g): all-*trans* (20.04); 9-*cis* (2,72); *cis*-isomers (3.04–3.07)		[35]
Loquat (fresh)[ *Eriobotrya japonica* (Thunb.) Lindl.]	HPLC-PDA-MS/MS: HPLC-MS: YMC C30 column (250 × 4.6 mm i.d., 5 μm particle)	β-cryptoxanthin (μg/100 g): all- *trans* (54.8–715.2); 9- or 90-*cis* (0.8); 13-or 130-*cis* (4.0–20.1); *cis*-5,6:50,60-diepoxy (1.8–3.5); 5,6:50,60-diepoxy (35.0–339.5); 5,8:50,60- or 5,6:50,80-diepoxy (1.8–34.8); *cis*-5,8:50,60- or 5,6:50,80-diepoxy (1.1–10.9); *cis*-5,6:50,60-diepoxy (1.9-12.1); 50,60-epoxy (11.5–109.4); 5,6-epoxy (19.0–213.9); 5,8-Epoxy (1.6–15.3) β-carotene (μg/100 g): all-*trans* (38.1–1441.5); 9-*cis* (1.6–18.0); 13-*cis* (5.0–45.9); 15-*cis* (0.7–4.8)		[36]
Mango (dried pulp)[ *Mangifera indica* L.]	HPLC: Polymeric C30 column (250 mm × 4.6 mm i.d., 5 μm particle)	Neoxanthin (μg/g): all- *trans* (0.44–0.71); *cis*-isomers (0.19–0.57)Violaxanthin (μg/g): all-*trans* (0.16–0.32); *cis*-isomers (0.10–4.70)Zeaxanthin (μg/g): all-*trans* (0.89–1.33); *cis*-isomers (0.72–0.96)Lutein (μg/g): 9- or 9’-*cis* (0.53–0.78)β-carotene (μg/g): all-*trans* (9.32–29.34); 13- or 13’-*cis* (0.78–3.79); 15- or 15’-*cis* (0.98–7.20); *cis*-isomers (0.35–0.70)		[37, 38]
Peach (fresh)[ *Prunus persica*(L.) Batsch]	HPLC: Polymeric C30 column (250 mm × 4.6 mm i.d., 5 μm particle)	β-cryptoxanthin (μg/g): all- *trans* (0.3); 13/13’-*cis* (0.1); 15-*cis* (0.1) β-carotene (μg/g): all-*trans* (2.2); 9-*cis* (0.3); 13-*cis* (0.5); 15-*cis* (trace)		[39]
Tree tomato (yellow) [ *Solanum betaceum* Cav.]	HPLC-MS: YMC C30 column (250 × 4.6 mm i.d., 5 μm particle)	β-carotene (% residual carotenoid): all- *trans* (61.1–85.5); 13-*cis* (284.2–518.6)ζ-carotene (% residual carotenoid): *cis*-isomer (46.5–83.9)		[13]
Broccoli (fresh)[ *Brassica oleracea* var. Italica]	HPLC: Polymeric C30 column (250 mm × 4.6 mm i.d., 5 μm particle)	β-carotene (μg/g): all- *trans* (29.2); 9-*cis* (5.0); 13-*cis* (3.3), 15-*cis* (1.9); *cis*-isomers (2.0)	[39]	
Maize (mutant, fresh)[ *Zea mays* L.]	HPLC: Spherisorb ODS-2 silica column (250 × 3.2 mm i.d., 5 μm particle)	ζ-carotene: di- *cis* (55.8) tri-*cis* (17.6–46.3)	[40]	
Maize (kernel) - 13 varieties [ *Zea mays* L.]	HPLC: Vydac218TP53 column (250 × 3.2 mm i.d.)	β-carotene (μg/100 g): all- *trans* (37–879); *cis*-isomers (<0.1–301)	[41]	
Pumpkin (fresh)[ *Curcurbita moschata* var. Orange]	HPLC: Polymeric C30 column (250 mm × 4.6 mm i.d., 5 μm particle)	β-carotene (μg/g): all- *trans* (61.6); 9-*cis* (2.5); 13-*cis* (2.7)	[15]	
Spinach (fresh)[ *Spinacia oleracea* L.]	HPLC: Polymeric C30 column (250 mm × 4.6 mm i.d., 5 μm particle)	β-carotene (μg/g): all- *trans* (311.9); 9-*cis* (38.6); 13-*cis* (24.5), 15-*cis* (trace); *cis*-isomers (22.5)	[39]	
Tomato (fresh)[ *Solanum lycopersicum* L.]	HPLC: Polymeric C30 column (250 mm × 4.6 mm i.d., 5 μm particle)	β-carotene (μg/g): all- *trans* (71.0); 9-*cis* (4.8); 13-*cis* (5.8)	[39]	

^a^ Ref.: References.

### 2.1. Carotenes

**Figure 1:**
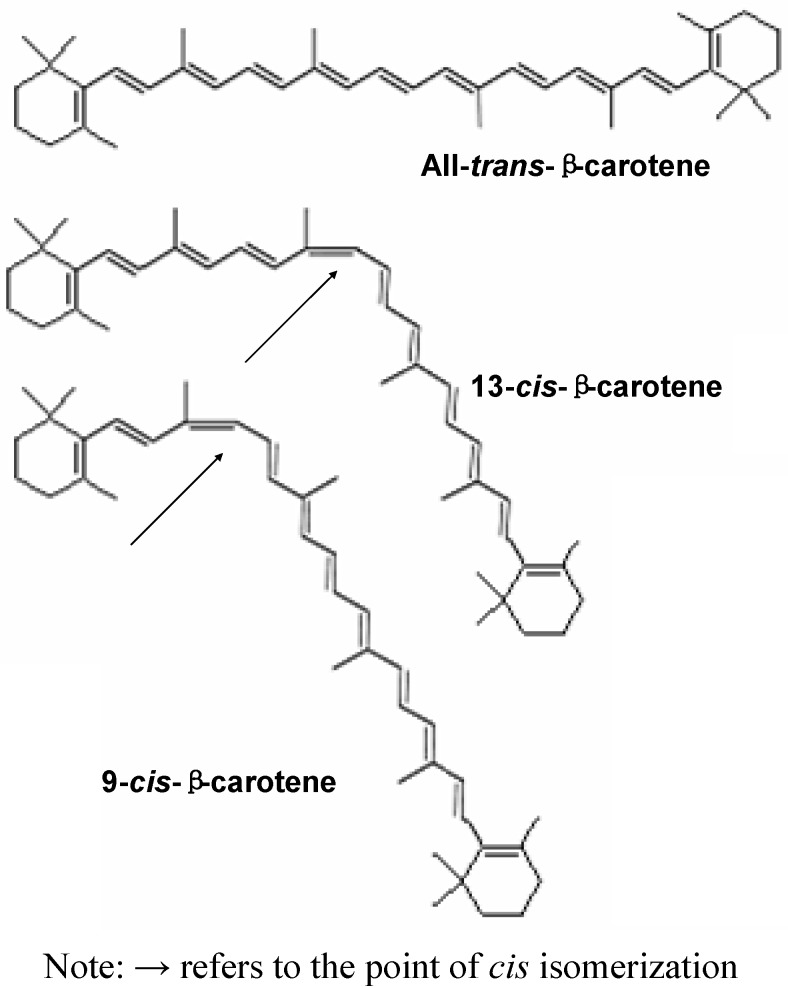
The structure of all-*trans*-β-carotene and its two geometric isomers [35,46].

Carotenes include several related compounds having the general formula C_40_H_56_. They are a simple type of carotenoid and occur in several isomeric forms, such as alpha (α), beta (β), gamma (γ), delta (δ), epsilon (ε), and zeta (ζ) [42]. Among the various carotenoids, α- and β-carotene are the two primary forms of carotenes. In human body, β-carotene is broken down by β-carotene dioxygenase in the mucosa of small intestine into two retinyl molecules, which is later reduced to vitamin A (retinol) [43]. Carotenes can be found in many dark green and yellow leafy vegetables and appear as fat soluble pigments, while β-carotene can be found in yellow, orange and red colored fruits and vegetables [44]. Naturally, β-carotene is mostly found as all-*trans* isomers and lesser as *cis*-isomers (Figure 1), with the relative abundances in the following order: all-*trans* > 9-*cis* > 13-*cis* > 15-*cis* [45]. 

All-*trans*-β-carotene is very unstable and can be easily isomerized into *cis*-isomers, when exposed to heat and light. Isomerization energy is involved in relocation of the single or double bond of one form of carotenoid into another [46,47]. A study has been carried out to determine the isomerization energy of carotenoids, especially neurosporene, spheroidene and spirilloxanthin [16], but the excited energy stages are not well understood. Besides, processing of fruit could result in significant *cis*-*trans* isomerization of β-carotene which was shown by the formation of 13-*cis*-β-carotene [48]. 

In regard to the effect of processing and isomerization of carotenoids in fruits and vegetables, 13-*cis*-β-carotene is the main product of geometric isomerization [49], 9-*cis*-β-carotene is formed when exposure to light [12,49], while 13-*cis*-α- and β-carotene isomers are formed during storage [50]. A study on the effect of β-carotene isomerization due to reflux heating has exhibited that degradation occurs to all-*trans*-β-carotene, with a significant increase in 13-*cis*-β-carotene [51]. Based on the structures of all-*trans*-β-carotenes, the double bonds can be relocated during heating and form several isomers (Figure 1) [46]. Marx *et al*. [52] have revealed that in pasteurized and sterilized samples, 13-*cis*-β-carotene was the only isomer formed during pasteurization and sterilization of carrot juice, while 9-cis-β-carotene was probably formed during blanching of sterilized carrot juice. Moreover, 9-*cis*- and 13-*cis*-β-carotenes were thought to originate independently from *cis* precursors by non-enzymatic isomerization of all-*trans* forms [53]. 

On the other hand, *cis*-β-carotene has been shown to isomerize into all-*trans*-isomer when heated and exposed to air [17]. It shows that isomerization of β-carotene occurs instead of degradation. The isomerization process was also known to occur when a crystaline β-carotene is heated at 90 °C and 140 °C in a nitrogen environment, which might be due to the partially melted β-carotene that has increased the probability of *cis*- to all-*trans*-β-carotene isomerization [17]. Carotene in all-*trans* form has higher bioavailability than its *cis* counterpart, while β-carotene and β-apo-12’-carotenal have the highest bioconversion rate at 100% and 120% (on a weight basis), respectively [54].

### 2.2. Lycopene

Lycopene is an unsaturated acyclic carotenoid with open straight chain hydrocarbon consisting of 11 conjugated and two unconjugated double bonds. Lycopene has no provitamin A activity due to the lack of terminal β-ionic ring as the basic structure for vitamin A [55]. Most of the lycopene occurs naturally in all-*trans* form [56]. The red color of lycopene is mainly due to many conjugated carbon double bonds, as it absorbs more visible spectrum compared to other carotenes [57]. Lycopene contains seven double bonds which can be isomerized to mono-*cis-* or poly-*cis*-isomers [58]. Based on the isomeric conformation of lycopene, 5-*cis*-lycopene was the most stable isomer, followed by all-*trans*- and 9-*cis*-lycopene [59]. Besides, 5-*cis*-lycopene has the lowest isomerization energy among other lycopene *cis*-isomers, and its very large rotational barrier restricts it to form all-*trans* structure [45]. More studies on isomerization energy are needed to explain the rationale on the conversion of all-*trans*-carotene to its *cis*-isomers by thermal processing, under low pH condition and exposure to light.

Lycopene *cis*-isomers are more soluble in oil or organic solvents than all-*trans*-lycopene [60]. There is dissimilarity between the isomerization of β-carotene and lycopene [61]. Lycopene isomerization occurs under the simulated gastric digestion, thermal processing and low pH [62], but the effect of these conditions on lycopene isomerization is unclear. Boileau *et al*. [56] reviewed that isomerization of lycopene was found to occur in human body due to the effect of gastric juice in the stomach. However, Blanquet-Diot *et al*. [59] reported that no *cis*-*trans* isomerization of lycopene has occurred using gastrointestinal tract model. Heating at 60 °C and 80 °C favored the isomerization of lycopene [63]. The formation of 9-*cis*-lycopene is more favorable at low pH condition while 13-*cis*-lycopene is the major degradation product formed from thermal processing [62]. 

The uptake of *cis*-lycopene by intestinal cells is known to exceed those of all-*trans*-lycopene, which was in agreement with the study by Tyssandier *et al*. [64] that *cis*-lycopene had greater bioaccessibility compared to its all-*trans* form. Lycopene *cis*-isomers also found to have greater bioactivity and bioavailability than their all-*trans* counterpart [65]. Besides, lycopene is less bioavailable than β-carotene and lutein [66]. Processing method could help to release the lycopene from the matrix in fruits and vegetables, and thus increases bioavailability [12]. 

### 2.3. Xanthophylls

Xanthophylls are the oxidized derivatives of carotenes. Xanthophylls, with a general chemical formula C_40_H_56_O_2_, contain hydroxyl groups and are more polar than carotenes [67]. In Nature, xanthophylls are found in the leaf of most plants and are synthesized within the plastids [68], which occur as yellow to red colored pigments. They are also considered accessory pigments, along with anthocyanins, carotenes, and sometimes phycobiliproteins [69].

Commonly found xanthophylls include lutein, zeaxanthin, and cryptoxanthin. In plant, violaxanthin, antheraxanthin and zeaxanthin participate in xanthophyll cycle, which involves the conversion of pigments from a non-energy-quenching form to energy-quenching forms [6]. Lutein is one kind of xanthophyll found abundently in fruits and vegetables [44,65]. It is a fat soluble compound and very stable in emulsion [66]. Although, lutein and zeaxanthin are isomers but they are not stereoisomers. In addition, lutein is one of the xanthophyll discovered in egg yolk [67]. As animals cannot produce xanthophylls, xanthophylls found in animals are known to be ingested from food [68].

The isomers of xanthophyll are not well studied. Since the development of the C_30_ HPLC analytical column, the determination of xanthophyll isomers is becoming a hot issue. Identification of xanthophyll isomers has been carried out using different polymeric columns [69]. Study also reported that *cis*-isomers of xanthophyll determined using a C_30_ stationary phase were relatively higher than accessed using C_18_ column [70]. Tóth and Szabolcs [71] had identified 9-*cis*- and 9’-*cis*-isomers of antheraxanthin, capsanthin, lutein and lutein epoxide in several higher plants. They found that 9-*cis*-isomers of antheraxanthin and lutein epoxide occurred without their 9’-*cis* counterparts in non-photosynthetic tissues. This could be explained by the non-stereoselective biosynthesis or stereomutation, while the 9-*cis* form is protected stereoselectively against photoisomerization. 

Isomers of violaxanthin namely, 9-*cis*-, 13-*cis*- and di-*cis*-violaxanthin have been identified in orange juice [72,73]. Besides, lutein epoxide has been identified in dandelion petal, with high amounts of the 9-*cis*- and 9’-*cis*-isomers, with the all-*trans* form as the major carotenoid [74]. Moreover, 13-*cis*-zeaxanthin was found as the major isomerization products of all-*trans* form, which was induced by light and temperature (35–39 °C) [75]. In one study by Kishimoto *et al*. [76], sixteen xanthophylls were isolated from the petals of chrysanthemum. These xanthophylls were mainly the isomers of violaxanthin, luteoxanthin, lutein, and also lutein epoxides. They also concluded that chrysanthemum petals have a unique carotenoid characteristic compared to the flowers of other species. Furthermore, Yahia *et al*. [77] reported that the saponified crude extract of mango fruit has all*-trans*-violaxanthin and 9-*cis*-violaxanthin present in the esterified form. In ripening fruit, esterification of xanthophylls occurs [10], but the mechanism and biosynthetic pathways of esterification are still to be explored. 

## 3. Carotenoid Pigments in Fruits and Vegetables

Carotenoids are widely distributed in the cellular tissues of plants [78]. The distribution of carotenoids in human tissues is originated from plant sources. Therefore, fruits and vegetables constitute the major source of carotenoids in human diet [79,80]. In plant, carotenoids are found as fat soluble and colored-pigments [81,82]. Carotenoids can be isolated from the grana of chloroplasts in the form of carotenoprotein complexes, which give various colors to the outer surfaces of the plants [83]. The visible colors of the plant are due to the conjugated double bonds of carotenoids that absorb light. The more number of double bonds results in the more absorbance of red color wavelength. The occurrences of carotenoids in plants are not as a single compound. Most of the carotenoids are bound with chlorophyll, and a combination of carotene-chlorophyll and xanthophyll-chlorophyll occurs often. The binding of carotenoids to chlorophylls can give rise to a variety of colors in plants, fruits and vegetables. However, as fruit matures, the chlorophyll content decreases, and results in colored-carotenoid pigments [84]. Besides, study had carried out to improve carotenoids color retention during ripening [85].

In nature, fruits have lesser xanthophyll contents compared to vegetables. Some fruits such as papaya (*Carica papaya* L.) and persimmon (*Diospyros sp.*) have high amount of xanthophylls (lutein and zeaxanthin), like that found in vegetables [44]. In fruits and vegetables, β-carotene is found to be bound to either chlorophylls or xanthophylls, forming chlorophyll-carotenoid complexes, which absorb light in the orange or red light spectrum and give rise to green, purple or blue coloration [86], These complexes could decrease the bioavailability of β-carotene and further weaken its bioefficacy for the conversion to vitamin A. However, this setback can be resolved by saponifying the plant extract to yield all*-trans*-β-carotene in a free-state form [77]. In vegetables, provitamin A carotenoids have lower bioavailability as compared to fruits [87], which may be due to their protein-complex structures in chloroplasts [54]. In this review, a comprehensive data for the typical carotenoids content in fruits and vegetables are given in Table 2 and Table 3, where the carotenoids contents in fruits and vegetables are summarized. The data from this compilation are useful for comparison of the ongoing study with other previous reports. 

### 3.1. Orange and yellow pigment carotenoids

Naturally occurring β-carotene, with 11 double bonds, is orange in color [55]. Takyi [83] reported β-carotene occurs as an orange pigment, while α-carotene is a yellow pigment, which can be found in fruits and vegetables. Yellow colored fruits that contain low or trace amounts of β-carotene are mainly from the genera *Ananas*, *Averrhoa*, *Citrus*, *Durio*, *Malus*, *Musa*, *Nephelium*, *Pyrus*, *Rubus* and *Vitis* or vegetables from the genera *Apium*, *Cucumis*, *Manihot*, *Vigna* and *Maranta* (Table 2 and Table 3). Besides, yellow maize (*Zea mays* L.) is a good source of β-carotene [88]. 

Several vegetables are known to contain β-carotene. For example, β-carotene is present in carrot, sweet potato and tomato which are from the genera of *Daucus*, *Ipome*a and *Solanum*, respectively. Carrot is the major contributor of β-carotene in the diet, along with green leafy vegetables. Rajyalakshmi *et al*. [89] reported that the β-carotene contents in 70 edible wild green leafy vegetables ranged from 0.4–4.05 mg per 100 gram edible portion. A few underutilized green leafy vegetables from India were also found to have 0.68–12.6 mg/100 g β-carotene [90]. Therefore, other than carotene-rich yellow-orange colored vegetables (e.g., carrot, pumpkin and sweet potato), green leafy vegetables are good sources of β-carotene. The β-carotene contents of some green leafy vegetable grown in the wild such as black nightshade (*Solanum nigrum*) and Mulla thotakura (*Amaranthus spinosus*) are comparable to carrot or sweet potato (Table 3). 

**Table 2:** Carotenoid contents (mg/100 g fresh weight) of some common fruits.

Taxonomy			Common name	α-Carotene	β-Carotene	Lycopene	References
Family	Genus	Species					
*Anacardiaceae*	*Mangifera*	*indica* L.	Mango	−	0.553	0.353	[91]
				−	1.71(0.95)	−	[90]
				0.017[0.001]	0.445[0.016]	−	[44]
		var. Black-gold		ND	0.615	ND	[65]
		var. Gedong		0.061(0.086)	3.267(2.075)	−	[92]
		var. Manalagi		ND	0.19(0.123)	−	[92]
		var. Indramayn		0.067(0.005)	1.606(0.166)	−	[92]
		var. Harum manis		0.055(0.001)	1.08(0.264)	−	[92]
		var. Golek		0.055(0.003)	1.237(0.626)	−	[92]
	*Spondias*	*dulcis* L.	Hog plum	−	0.201	0.364	[91]
*Actinidiaceae*	*Actinidia*	*deliciosa* C.F.Liang.& A.R.Ferguson.	Kiwifruit				
		var. Hayward		ND	0.074[0.021]	ND	[93]
		var. Zespri gold		ND	0.092[0.008]	ND	[93]
*Bromeliaceae*	*Ananas*	*comosus* (L.) Merr.	Pineapple	ND	0.056[0.005]	ND	[93]
				ND	0.17	ND	[94]
*Caricaceae*	*Carica*	*papaya* L.	Papaya	ND	0.23-1.981	1.477-5.75	[65,94,95]
				−	1.05(0.44)	−	[90]
				ND	0.276[0.245]	−	[44]
		var. Fruit tower		ND	0.409[0.027]	2.481[0.692]	[93]
		var. Sun rise		ND	1.981[0.059]	1.477[0.302]	[93]
		var. Yellow sweet		ND	1.048[0.026]	1.987[0.851]	[93]
		var. Hawaiian		ND	0.5	1.7	[94]
*Cucurbitaceae*	*Citrullus*	*lanatus* (Thunb.) Matsum. & Nakai	Watermelon	0-0.76	0.14-6.806	0.071-11.389	[65,91,94,95]
				ND	0.59[0.033]	6.184[0.152]	[93]
*Ebenaceae*	*Diospyros*	*sp.*	Persimmon	−	0.253	−	[44]
				ND	0.129[0.003]	0.415[0.013]	[93]
*Ericaceae*	*Vaccinium*	*spp.*	Blueberries	ND	0.035	ND	[44]
				ND	0.027[0.005]	ND	[93]
*Malvaceae*	*Durio*	*zibethinus* L.	Durian	0.006	0.023	−	[44]
*Moraceae*	*Artocarpus*	*heterophyllus* Lam.	Jackfruit	ND	0.026-0.36	0.037	[65,91,94,96]
				−	0.16(0.06)	−	[90]
*Musaceae*	*Musa*	*spp.*	Banana	0.005[0.005]	0.021[0.014]	−	[44]
				0.058[0.007]	0.058[0.006]	ND	[93]
		*paradisiaca* L.var. Ambon	Banana	−	0.097	0.114	[91]
		*sapientum* Linn.					
		var. Emas		−	0.04	−	[65]
		var. Tanduk		−	0.092	−	[65]
*Myrtaceae*	*Psidium*	*guajava* L.	Guava	−	0.001	0.114	[91]
				−	0.001 (0.0001)	−	[90]
		var. Pink	Pink guava	−	−	5.4	[95]
				ND	0.359[0.015] 5.027[0.08]	2.307[0.058] 4.383[0.371]	[93]
*Oxalidaceae*	*Averrhoa*	*carambola* L.	Starfruit	ND	0.028-0.042	0-0.042	[65,91,96]
				ND	ND	ND	[93]
*Passifloraceae*	*Passiflora*	*edulis* Sims	Passion fruit	0.035	0.53	−	[44]
				ND	0.156[0.02]	0.057[0.003]	[93]
*Rosaceae*	*Eriobotrya*	*japonica*(Thunb.) Lindl.	Loquat	−	0.207	−	[97]
	*Fragaria*	*ananassa* Duchesne	Strawberry	0.005	−	−	[44]
	*Malus*	*domestica* Borkh.	Apple	0.001-0.03	0.031-0.072	0.209	[96,98,99]
		var. Fuji		ND	0.036[0.003]	ND	[93]
*Rosaceae*	*Prunus*	*armeniaca* L.	Apricot	ND	2.554	0.005	[44]
		*salicina* Lindl.	Nectarine				
		var. Red Jim		−	0.073(0.016)	−	[100]
		var. August red		−	0.128(0.005)	−	[100]
		var. Spring bright		−	0.085(0.006)	−	[100]
		var. May glo		−	0.058(0.005)	−	[100]
		var. September red		−	0.131(0.023)	−	[100]
		*persica* (L.) Batsch	Peach	0.001[0.001]	0.097[0.013]	−	[44]
		var. Summer sweet		−	0.04(0.01)	−	[100]
		var. Snow king		−	0.008(0.002)	−	[100]
		var. Snow giant		−	0.006(0.001)	−	[100]
		var. Champagne		−	0.007(0.001)	−	[100]
		var. September snow		−	0.004(0.001)	−	[100]
		var. Hakuto		ND	0.048[0.032]	ND	[93]
		var. Kanto 5 go		ND	0.036[0.006]	ND	[93]
		var. Mochizuki		ND	ND	ND	[93]
		var. Nishiki		ND	0.16[0.005]	ND	[93]
		var. Ogonto		ND	0.121[0.008]	ND	[93]
		*domestica* L.	Plum	−	0.098	−	[44]
		var. Red		ND	0.127	ND	[65]
		var. Wickson		−	0.04(0.004)	−	[100]
		var. Black Beaut		−	0.188(0.017)	−	[100]
		var. Red Beaut		−	0.064(0.012)	−	[100]
		var. Santa Rosa		−	0.049(0.012)	−	[100]
		var. Angeleno		−	0.057(0.009)	−	[100]
		var. Ponteroza		ND	0.218[0.019]	ND	[93]
		var. Soldam		ND	0.439[0.029]	ND	[93]
*Rosaceae*	*Prunus*	*spp.*	Cherry	ND	ND	ND	[94]
				−	0.14(0.06)	−	[90]
				−	0.028	−	[44]
		var. Domestic		0.018(0.004)	0.071(0.004)	ND	[93]
		var. USA		ND	0.037(0.004)	ND	[93]
	*Pyrus*	*sp.*	Pear	0.006	0.027	ND	[44]
	*Rubus*	*sp.*	Raspberry	0.012	0.008	−	[44]
*Rutaceae*	*Citrus*	*aurantium* L.	Orange	−	0.17(0.08)	−	[90]
		*maxima* Merr.	Pummelo	0.014	0.32	−	[44]
		*microcarpa* Bunge	Musk lime	−	0.012	−	[65]
		*nobilis* L.	Orange	−	0.025	−	[65]
		*paradisiaca* Macfad.	Grapefruit				
		var. Star ruby		ND	0.452[0.019]	1.869[0.654]	[93]
		var. Pink		0.005[0.005]	0.603[0.152]	−	[44]
				−	−	3.36	[95]
		var. White		0.008	0.014	−	[44]
		*sinensis* (L.) Osbeck	Orange	0.016	0.051	−	[44]
		var. Navel		0.019[0.002]	0.139[0.014]	ND	[93]
		var. Valencia		0.015[0.001]	0.051[0.004]	ND	[93]
		*reticulata* Blanco	Mandarin orange	−	0.081	−	[65]
				ND	0.03	ND	[94]
*Sapindaceae*	*Nephelium*	*lappaceum* L.	Rambutan	−	ND	0.148	[91]
*Vitaceae*	*Vitis*	*vinifera* Linnaeus	Grape	−	0.039	−	[44]
		var. Deraware		ND	0.058[0.004]	ND	[93]
**Taxonomy **			**Common name**	**β-cryptoxanthin**	**Lutein**	**Zeaxanthin**	**References**
**Family**	**Genus**	**Species**					
*Anacardiaceae*	*Mangifera*	*indica* L.	Mango	0.137	−	−	[91]
				0.011[0.009]	−	−	[44]
		var. Black-gold		ND	ND	−	[65]
	*Spondias*	*dulcis* L.	Hog plum	0.309	−	−	[91]
*Actinidiaceae*	*Actinidia*	*deliciosa* L.	Kiwifruit				
		var. Hayward		ND	0.153(0.005)	ND	[93]
		var. Zespri gold		ND	0.156(0.005)	0.113(0.006)	[93]
*Bromeliaceae*	*Ananas*	*comosus* (L.) Merr.	Pineapple	0.089	ND	ND	[93]
*Caricaceae*	*Carica*	*papaya* L.	Papaya	0.18-3.182	0.016-0.063	0.165-0.564	[65,91,95]
				0.076[0.225]	0.075c	−	[44]
		var. Fruit tower		0.725[0.012]	0.016[0.001]	0.165[0.001]	[93]
		var. Sun rise		3.182[0.117]	0.063[0.001]	0.564[0.01]	[93]
		var. Yellow sweet		1.629[0.064]	0.029[0.001]	0.303[0.007]	[93]
		var. Hawaiian		−	−	−	[94]
*Cucurbitaceae*	*Citrullus*	*lanatus* (Thunb.) Matsum. & Nakai	Watermelon	0.09-0.48	0, 0.017c	ND	[65,91,95]
				ND	ND	ND	[93]
*Ebenaceae*	*Diospyros*	*sp.*	Persimmon	1.45	0.834c	0.49	[44]
				0.52[0.02]	ND	0.238[0.01]	[93]
*Ericaceae*	*Vaccinium*	*spp.*	Blueberries	−	−	−	[44]
				0.011[0.006]	0.042[0.011]	ND	[93]
*Malvaceae*	*Durio*	*zibethinus* L.	Durian	ND	−	−	[44]
*Moraceae*	*Artocarpus*	*heterophyllus* Lam.	Jackfruit	0.017-0.036	0.095	−	[65,91,96]
*Musaceae*	Musa	*spp.*	Bananas	ND	NDc	−	[44]
				ND	0.113(0.008)	ND	[93]
		*paradisiaca* L.var. Ambon		0.003	−	−	[91]
*Myrtaceae*	*Psidium*	*guajava* L.	Guava	0.012, 0.464	0.044	ND	[91,95]
		var. Pink	Pink guava	0.012[0.003], 0.464[0.015]	0.044[0.002]	ND	[93]
*Oxalidaceae*	*Averrhoa*	*carambola* L.	Starfruit	0.036-1.066	0.066	ND	[65,91,96]
				ND	ND	ND	[93]
*Passifloraceae*	*Passiflora*	*edulis* Sims.	Passion fruit	0.046	−	−	[44]
				0.027[0.001]		0.042[0.002]	[93]
*Rosaceae*	*Eriobotrya*	*japonica*(Thunb.) Lindl.	Loquat	0.518	−	−	[97]
	*Fragaria*	*ananassa* Duchesne	Strawberry	−	−	−	[44]
	*Malus*	*domestica* Borkh.	Apple	0.001-0.106	0.017	0.0019	[91,96,99]
		var. Fuji		ND	ND	ND	[93]
	*Prunus*	*armeniaca* L.	Apricot	ND	−	−	[44]
		*salicina* Lindl.	Nectarine				
		var. Red Jim		0.014(0.005)	−	−	[100]
		var. August red		0.014(0.003)	−	−	[100]
		var. Spring bright		0.021(0.002)	−	−	[100]
		var. May glo		0.008(0)	−	−	[100]
		var. September red		0.015(0.006)	−	−	[100]
		*persica* (L.) Batsch	Peach	0.024	0.057^c^	−	[44]
		var. Summer sweet		0.012(0)	−	−	[100]
		var. Snow king		ND	−	−	[100]
		var. Snow gaint		ND	−	−	[100]
		var. Champagne		ND	−	−	[100]
		var. September snow		ND	−	−	[100]
*Rosaceae*	*Prunus*	*persica* (L.) Batsch	Peach				
		var. Hakuto		ND	ND	ND	[93]
		var. Kanto 5 go		0.283[0.003]	ND	0.51[0.015]	[93]
		var. Mochizuki		0.081[0.011]	ND	0.028[0.002]	[93]
		var. Nishiki		0.074[0.003]	0.051[0.005]	0.116[0.005]	[93]
		var. Ogonto		0.025[0.008]	0.029[0.002]	0.104[0.002]	[93]
		*domestica* L.	Plum	0.016	−	−	[44]
		var. Red		0.04	0.149	−	[65]
		var. Wickson		0.05(0.01)	−	−	[100]
		var. Black Beaut		0.13(0.01)	−	−	[100]
		var. Red Beaut		0.03(0.01)	−	−	[100]
		var. Santa Rosa		0.07(0.03)	−	−	[100]
		var. Angeleno		0.03(0)	−	−	[100]
		var. Ponteroza		0.05[0.008]	0.133[0.024]	0.049[0.006]	[93]
		var. Soldam		0.077[0.009]	0.207[0.011]	0.026[0.002]	[93]
		*spp.*	Cherry	−	−	−	[44]
		var. Domestic		0.021[0.001]	0.112[0.008]	0.042[0.005]	[93]
		var. USA		0.014[0.002]	0.091[0.004]	0.027[0.001]	[93]
*Rutaceae*	*Citrus*	*maxima* Merr.	Pummelo	0.103	−	−	[44]
		*paradise* Macfad.	Grapefruit				
		var. Star ruby		ND	ND	ND	[93]
		var. Pink		0.012[0.009]	−	−	[44]
		var. White		−	−	−	[44]
		*nobilis* L.	Orange	−	0.275	ND	[91]
		*sinensis* (L.) Osbeck	Orange				
		var. Navel		0.462[0.031]	0.059[0.006]	0.164[0.013]	[93]
		var. Valencia		0.278[0.001]	0.071[0.002]	0.019[0.001]	[93]
				0.122	0.187c	−	[44]
*Sapindaceae*	*Nephelium*	*lappaceum* L.var. Deraware	Rambutan	ND	−	−	[91]
				ND	0.103[0.014]	0.028[0.004]	[93]

^a ^ND, Not detected; − data not available; var., variety; ^b ^mean(standard deviation), mean[standard error]; ^c ^content of lutein + zeaxanthin

**Table 3:** Carotenoid contents (mg/100 g fresh weight) of common leafy and non-leafy vegetables.

Taxonomy			Common name	α-Carotene	β-Carotene	Lycopene	References
Family	Genus	Species					
***Leafy Vegetables***							
*Alliaceae*	*Allium*	*fistulosum* L.	Spring onion leaves	−	1.28	−	[65]
		*sativum* L.	Garlic leaves	−	5.0	−	[42]
		*cepa* L.	Onion leaves	−	4.9(0.15)	−	[90]
*Apiaceae*	*Apium*	*graveolens* L.	Celery	ND	0.77	ND	[100]
				ND	0.15	−	[43]
	*Coriandrum*	*sativum* L.	Coriander leaves	ND	3.17	ND	[65]
			Coriander	−	4.8(0.16)	−	[90]
	*Foeniculum*	*vulgare* Mill.	Fennel common	−	4.4	−	[101]
*Amaranthaceae*	*Amaranthus*	*spp.*	Amaranth	−	1.96-8.6	−	[42,101,102]
		*spinosus* L.	Mulla thotakura	−	10.9(1.25)	−	[90]
		*sp.*	Yerramolakakaura	−	11.9(1.48)	−	[90]
	*Spinacia*	*oleracea* L.	Spinach	ND	3.177, 36.53(6.4)	ND	[65,103]
		var. Red		ND	5.088	ND	[65]
				−	1.1(0.36)	−	[90]
				ND	5.597[0.561]	−	[44]
*Asteraceae*	*Lactuca*	*sativa* L.	Lettuce	ND	0.097	ND	[65]
				−	1.4(0.28)	−	[90]
		var. Cos or Romaine		ND	1.272	−	[44]
		var. Iceberg		0.002	0.192[0.069]	−	[44]
*Brassicaceae*	*Brassica*	*juncea* (L.) Czern.	Chinese mustard leaves	ND	2.93	ND	[65]
		*oleracea* L.					
		var. Acephala	Kale	ND	9.23	ND	[44]
		var.Alboglabra	Chinese kale	ND	4.09	ND	[65]
		var. Capitata	Cabbage	ND	0.01-3.02	ND	[44,101]
		var. Chinensis		−	2.703	−	[65]
		var. Pekinensis		−	0.01(0.01)	−	[104]
		*papaya* L.	Papaya leaves	0.424(0.355)	5.229(2.195)		[92]
		*aquatica* Forssk.	Swamp cabbage	ND	1.895	ND	[61]
			Water spinach	0.014(0.026)	2.73 (1.013)	−	[92]
*Cucurbitaceae*	*Momordica*	*Charantia* Descourt.	Bitter melon leaves	−	3.4	−	[101]
*Euphorbiaceae*	*Manihot*	*esculenta* Crantz	Cassava leaves	0.038(0.054)	9.912(2.503)	−	[92]
*Fabaceae*	*Sesbania*	*grandiflora* (L.) Poiret	Sesbania	ND	13.61, 13.28(3.2)	ND	[65,103]
	*Trigonella*	*foenum-graecum* L.	Fenugreek	−	9.2(1.48), 12.13(4.1)	−	[91,103]
*Lamiaceae*	*Mentha*	*arvensis* L.	Pudina	−	4.3(2.0)	−	[90]
*Meliaceae*	*Azadirachta*	*indica* L.	Neem tree leaves	−	0.92	−	[101]
*Moringaceae*	*Moringa*	*oleifera* Lam.	Drumstick leaves	ND	5.2, 7.54	ND	[65,102]
				−	19.7(5.55), 22.89(6.8)	−	[91,103]
*Phyllanthaceae*	*Sauropus*	*androgynus* L.	Sweet shoot leaves	ND	13.35	ND	[65]
				1.335(0.878)	10.01(2.189)	−	[92]
*Solanaceae*	*Solanum*	*nigrum* L.	Black nightshade	ND	7.05	ND	[65]
*Rutaceae*	*Murraya*	*koenigii* (L.) Sprengel	Curry leaves	−	7.1(2.36)	−	[90]
***Non-leafy Vegetables***							
*Alliaceae*	*Allium*	*schoenoprasum* L.	Chive	ND	0.83, 3.51	ND	[42,65]
*Apiaceae*	*Daucus*	*carota* L.	Carrot	3.41-6.2	6.5-21	ND	[44,65,93]
*Araceae*	*Colocasia*	*esculenta* (L.) Schott	Taro	−	−	−	−
*Asparagaceae*	*Asparagus*	*officinalis* L.	Asparagus	0.012	0.493	−	[44]
*Brassicaceae*	*Brassica*	*oleracea* L.					
		var. Calabrese	Broccoli	−	0.898	−	[97]
				0.001[0.001]	0.779[0.19]	−	[44]
*Brassicaceae*	*Brassica*	var. Italica Plenck.		−	0.81(0.2)	−	[103]
		var. Gemmiferae	Brussels sprout	ND	0.14	ND	[42]
				0.006	0.45[0.057]	−	[44]
				−	0.14(0.02)	−	[104]
		var. Botrytis	Cauliflower	−	0.08	−	[42]
				−	0.08(0.03)	−	[104]
				−	6.5(1.46)	−	[90]
*Convolvulaceae*	*Ipomea*	*batatas* (L.) Lam	Sweet potato	0.002	0.058, 9.18	ND	[44,92]
				−	1.87(0.14)	−	[90]
				ND	9.18[1.272]	−	[44]
*Cucurbitaceae*	*Coccinea*	*grandis* (L.) J. Voigt	Ivy gourd	−	3.2-4.1	−	[42]
	*Cucumis*	*sativus* L.	Cucumber	0.008	0.031-0.14	ND	[44]
				ND	ND	ND	[94]
	*Cucurbita*	*maxima* Duch.	Pumpkins	0.03-7.5	0.06-14.85	ND	[65,94]
		(12 varieties)		0-7.5	1.4-7.4	−	[105]
		*minima* L.		−	1.16(0.057)	−	[90]
		*moschata* Duch.		−	9.29(7.5)	−	[106]
		(4 varieties)		0.98-5.9	3.1-7.0	−	[105]
		*pepo* L. (5 varieties)		0.03-0.17	0.06-2.3	−	[105]
	*Momordica*	*charantia* Descourt.	Bitter gourd	ND	ND	ND	[94]
*Euphorbiaceae*	*Manihot*	*esculenta* Crantz	Cassava	ND	0.008	−	[44]
		var. Monroe		ND	0.52	ND	[94]
		var. Beqa		ND	0.43	ND	[94]
		var. Common		ND	<0.02	ND	[94]
		*utilissima* Pohl.	Tapioca shoot	ND	5.72	ND	[65]
	*Phaseolus*	*vulgaris* L.	French bean	ND	0.24	ND	[65]
		var. Red	Common Bean	0.28	0.8	ND	[94]
		var. Yellow		ND	ND	−	[94]
		var. French		0.72	0.78	ND	[94]
	*Vigna*	*unguiculata* (L.) Walp.					
		subsp. *unguiculata*	Cow pea	−	−	−	[107]
		subsp. *sesquipedalis*	Long bean	ND	0.41-0.57	ND	[65]
*Malvaceae*	*Abelmoschus*	*esculentus* (L.) Moench	Okra	0.028	0.43	−	[44]
*Marantaceae*	*Maranta*	*arundinacea* L.	Arrowroot	ND	0.01	−	[44]
*Poaceae*	*Zea*	*mays* L.	Maize	−	0.014	−	[44]
		(13 varieties)		0.003-0.086 (0-0.009)	0.037-0.879 (0-0.028)	−	[92]
*Solanaceae*	*Capsicum*	*annuum* L.					
		var. Cayenne	Chilies	3.41	0.47-6.77	ND	[65]
		var. Grossa	Capsicum	−	1.13(0.8)	−	[90]
				ND	0.27	ND	[65]
		*sp.*	Pepper	0.022-0.059	0.2-2.38	ND	[44]
				−	0.11 (0.04)	−	[91]
	*Solanum*	*betaceum* Cav.	Tree tomato	ND	0.6	ND	[65]
		*lycopersicum* L.	Tomatoes	2.5	0.365-1.3	0.009-2.0	[65,94,95]
				−	0.62(0.19)	−	[90]
		*melongena* L.	Red eggplant	ND	ND	ND	[94]
		*tuberosum* L.	Potato	−	0.006	−	[44]
**Taxonomy **			**Common name**	**β-Cryptoxanthin**	**Lutein**	**Zeaxanthin**	**References**
**Family**	**Genus**	**Species**					
***Leafy Vegetables***								
*Alliaceae*	*Allium*	*fistulosum* Linnaeus	Spring onion leaves	ND	0.323	−	[65]
*Amaranthaceae*	*Spinacia*	*oleracea* L.	Spinach	−	77.58(6.6)	1.51(0.4)	[103]
*Apiaceae*	*Apium*	*graveolens* L.	Celery	ND	0.23^c^	0.003	[44]
	*Spinacia*	*oleracea* L.	Spinach	ND	4.175	−	[65]
		var. Red		ND	2.047	−	[65]
				ND	11.938^c^	−	[44]
*Asteraceae*	*Lactuca*	*sativa* L.	Lettuce	ND	0.073	−	[65]
		var. Cos or Romaine		ND	2.635^c^	−	[44]
		var. Iceberg		ND	0.352^c^	−	[44]
*Brassicaceae*	*Brassica*	*juncea* (L.) Czern.	Chinese mustard	ND	1.02	−	[65]
		*oleracea* L.					
		var. Acephala	Kale	ND	39.55^c^	−	[44]
		var. Alboglabra	Chinese kale	ND	1.54	−	[65]
		var. Capitata	Cabbage	ND	0.02, 0.31^c^	−	[44,101]
		*rapa* L.	Chinese cabbage				
		var. Chinensis		−	2.703	−	[65]
		var. Pekinensis		−	0.02(0.01)	−	[104]
*Convolvulaceae*	*lpomoea*	*aquatica* Forssk.	Swamp cabbage	ND	0.335	−	[65]
*Fabaceae*	*Sesbania*	*grandiflora* (L.) Poiret	Sesbania	ND	20.21, 16.9(3.7)	0.57(0.7)	[65,103]
*Moringaceae*	*Moringa*	*oleifera* Lam.	Drumstick leaves	ND	7.13, 50.4(0.8)	4.13(0.7)	[102,103]
*Phyllanthaceae*	*Sauropus*	*androgynus* L.	Sweet shoot leaves	ND	29.91	−	[65]
*Rutaceae*	*Murraya*	*koenigii* (L.) Sprengel	Curry leaves	ND	5.25	−	[65]
*Solanaceae*	*Solanum*	*nigrum* L.	Black nightshade	ND	2.89	−	[65]
***Non-leafy Vegetables***							
*Alliaceae*	*Allium*	*schoenoprasum* L.	Chive	ND	1.08	−	[42,65]
*Apiaceae*	*Daucus*	*carota* L.	Carrot	ND	ND	−	[44]
*Araceae*	*Colocasia*	*esculenta* (L.) Schott	Taro	−	0.16	0.006	[107]
*Brassicaceae*	*Brassica*	*oleracea* L.					
		var. Calabrese	Broccoli	ND	1.28, 2.45^c^		[44,97]
		var. Italica Plenck		−	0.68(0.22)	−	[104]
		var. Gemmiferae	Brussels sprout	ND	0.43	−	[42]
				ND	1.59^C^	−	[44]
				−	0.43(0.06)	−	[104]
		var. Botrytis	Cauliflower	−	0.13	−	[42]
				−	0.05(0.02)	−	[104]
*Convolvulaceae*	*Ipomea*	*batatas* (L.) Lam	Sweet potato	ND	ND	−	[44]
*Cucurbitaceae*	*Coccinea*	*grandis* (L.) J. Voigt	Ivy gourd	−	0.99	ND	[107]
	*Cucumis*	*sativus* L.	Cucumber	−	0.544	0.009	[107]
	*Cucurbita*	*maxima* Duch.	Pumpkins	ND	0.94-17.0	0.278	[65,107]
		(12 varieties)		−	0.8^c^-17.0^c^	−	[105]
		*minima* L.		−	1.16(0.057)	−	[90]
		*moschata* Duch.		−	9.29(*7.5*)	−	[106]
		(4 varieties)		−	0.08^c^-1.1^c^	−	[105]
		*pepo* L. (5 varieties)		−	0^c^-1.8^c^	−	[105]
*Euphorbiaceae*	*Manihot*	*esculenta* Crantz	Cassava	ND	−	−	[44]
		var. Monroe		−	−	−	[94]
		var. Beqa		−	−	−	[94]
		var. Common		−	−	−	[94]
		*utilissima* Pohl.	Tapioca shoot	ND	1.68^c^	−	[65]
*Fabaceae*	*Phaseolus*	*vulgaris* L.	French bean	ND	0.171-0.46	0.02	[65,107]
	*Vigna*	*unguiculata* (L.) Walp.					
		subsp. *unguiculata*	Cow pea	−	0.24	0.009	[107]
		subsp. *sesquipedalis*	Long bean	ND	0.3-0.42	−	[65]
*Malvaceae*	*Abelmoschus*	*esculentus* (L.) Moench	Okra	−	0.347	0.008	[44]
*Marantaceae*	*Maranta*	*arundinacea* L.	Arrowroot	ND	−	−	[44]
*Poaceae*	*Zea*	*mays* L. (13 varieties)	Maize	0.037-0.988 (0.001-0.015)	0-2.047 (0-0.075)	0.173-2.07 (0.004-0.073)	[92]
*Solanaceae*	*Capsicum*	*annum* L.					
		var. Cayenne	Chilies	1.75	0.39-1.902	0.063	[65,107]
		var. Grossa	Capsicum	−	0.425	0.005	[107]
		*spp.*	Pepper	2.21	0.22	−	[44,65]
	*Solanum*	*betaceum* Cav.	Tree tomato	1.24	ND	−	[65]
		*lycopersicum* L.	Tomatoes	ND	0.13-0.289	0.014	[65,107]
		*melongena* L.	Red eggplant	−	0.065-1.8	0.005-0.016	[107]
		*tuberosum* L.	Potato	−	−	−	−
	*Phaseolus*	*vulgari* L.	French bean	ND	0.171-0.46	0.02	[65,107]

^a ^ND, Not detected; − data not available; var., variety; ^b ^mean(standard deviation), mean[standard error]; ^c ^content of lutein + zeaxanthin

Orange colored fruits such as apricot (*Prunus armeniaca* L.), grapefruit (Citrus *paradise* Macfad.), mango (*Mangifera indica* L.), papaya (*Carica papaya* L.), persimmon (*Diospyros sp.*), pink guava (*Psidium guajava* L. var. Pink) and watermelon [*Citrullus lanatus* (Thunb.) Matsum. & Nakai] are rich in β-carotene. Khoo *et al*. [108] reported that orange colored underutilized fruits contained high amount of β-carotene. Although papaya is orange in color, certain cultivars have shown to contain low β-carotene [93,94]. Furthermore, Levy *et al*. [98] reported some of the orange colored fruits had low amount of β-carotene. 

Naturally, most of xanthophylls are yellow-orange colored pigments, especially lutein and zeaxanthin which can be found in most of the fruits and vegetables [82]. As lutein can absorb blue light, it appears as yellow color; while zeaxanthin appears yellow-orange color. Cryptoxanthins are other types of yellow-orange colored carotenoids. Takyi [82] reported α-cryptoxanthin appears as yellow colored pigment, while β-cryptoxanthin is orange in color. As shown in Table 3, lutein is found to be in higher amounts in green leafy and yellow colored non-leafy vegetables as compared to fruits. 

Green leafy vegetables that contain high amount of xanthophylls are mainly from the genera of *Brassica*, *Coriandrum*, *Lactuca*, *Moringa*, *Murraya*, *Sauropus*, *Sesbania*, *Solanum* and *Spinacia*, while the non-leafy vegetables are from the genera of *Allium*, *Brassica*, *Capsicum*, *Cucurbita* and *Zea* (Table 3). These green vegetables contain mainly lutein and zeaxanthin [44,65]. Kale (*Brassica oleracea* L. var. Acephala), lettuce (*Lactuca sativa* L. var. Cos or Romaine), Sesbania (*Sesbania grandiflora* L. Poiret), spinach (*Spinicia oleracea* L. var. Red) and sweet shoot leaves (*Sauropus androgynus* L.) are the example of the leafy vegetables that have high lutein content (Table 3); while other lutein-rich non-leafy vegetables are red eggplant (*Solanum melongena* L.), chili (*Capsicum annum* L. var. Cayenne), ivy gourd [*Coccinea grandis* (L.) J. Voigt], and pumpkin (*Cucurbita maxima* Duch.) [107]. 

Muzhingi *et al*. [88] reported that 36 genotypes of yellow maizes (*Zea mays* L.) contained lutein and zeaxanthin, whereas saponification significantly decreased the xanthophyll contents. In some cases, zeaxanthin-rich fruits [e.g., papaya (*Carica papaya* L.) and persimmon (*Diospyros sp.*)] and zeaxanthin-rich non-leafy vegetables [e.g. pumpkin (*Cucurbita* *maxima* Duch.) and maize (*Zea mays* L.)] were found to have high amount of β-carotene (Table 2 and Table 3). Cryptoxanthin is another yellow colored carotenoid, which is closely related to carotene [9,82]. Cryptoxanthin has approximately half of provitamin A activity as compared to β-carotene [109]. Cryptoxanthins have been identified in various types of fruits and vegetables [9,82,110]. Besides, the level of β-cryptoxanthin is high in fruits such as papaya (*Carica papaya* L.), persimmon (*Diospyros sp.*) and starfruit (*Averrhoa carambola* L.) (Table 2) and non-leafy vegetables such as chili (*Capsicum annum* L. var. Cayenne), maize (*Zea mays* L.), pepper (*Capsicum sp.*) and tree tomato (*Solanum betaceum* Cav.) (Table 3). 

### 3.2. Red pigment carotenoids

Lycopene is one of the naturally occurring red colored carotenoids [58]. The all*-trans-*isomer of lycopene is the most predominant geometrical isomer in fruits and vegetables [111]. Lycopene has two more double bonds than β-carotene, hence it appears red. Beside lycopene, δ-carotene pigment is red-orange in color, while astaxanthin is a red colored pigment [82]. 

This review shows that red lycopene pigment is abundant in fruits such as papaya (*Carica papaya* L.), pink grapefruit (*Citrus paradise* Macfad. var. Pink), pink guava (*Psidium guajava* L. var. Pink) and watermelon [*Citrullus lanatus*(Thunb.) Matsum. & Nakai] [93]. For non-leafy vegetables, USDA database [112] showed that raw red cabbage (*Brassica oleracea* var. Capitata) and boiled asparagus (*Asparagus officinalis* L.) contained 20 and 30 μg lycopene per 100 g edible portions. Red colored pigments in fruits and vegetables are believed to be originated from lycopene, which might also account for xanthophylls [113,114,115] and anthocyanins [116,117]. 

In fruits, dry persimmon (*Diospyros sp.*) contains the highest amount of lycopene (53.21 mg/100 g dry weight), which is two times higher than dry tomato [118]. A review by Bramley [95] has shown that pink guava and watermelon had comparable amounts of lycopene, which are even higher than fresh tomato (Table 2 and Table 3). Lycopene content in tomato products such as tomato ketchup is 5.5-time higher than in fresh ripe tomato [44]. Lycopene content in fresh tomato is influenced by the cultivars, agricultural practices, maturity and environmental factors [61]. Besides, mutant tomatoes have an almost two-fold increase in lycopene content [119]. 

## 4. Conclusions

Carotenoids are colorful pigments found in fruits and vegetables. The geometric isomers of carotenoids are present in all-*trans* and *cis* forms, together with carotenoid epoxides. Although all-*trans*-isomer is the major form of carotenoid, the *cis* isomers are available in small quantities. Heating and thermal processing could increase the amount of carotenoid *cis*-isomers. The degradation of carotenoids in fruits and vegetables is a major issue due to carotenoid loss. More attention should be given to the control of carotenoid geometry isomer degradation, and to improve the quality of dietary carotenoids. The color changes during geometric isomerization of carotenoids, thermal processing and even fruit ripening have been widely studied. However, there is still lack of information on the chemical and kinetic pathways of color changes during carotenoid degradation and isomerization. In the future, more studies are needed to focus on the isomerization of carotenoids in relation to the colorful pigments in the biodiversity of fruits and vegetables. 
